# Process engineering of pH tolerant *Ustilago cynodontis* for efficient itaconic acid production

**DOI:** 10.1186/s12934-019-1266-y

**Published:** 2019-12-12

**Authors:** Hamed Hosseinpour Tehrani, Katharina Saur, Apilaasha Tharmasothirajan, Lars M. Blank, Nick Wierckx

**Affiliations:** 1 iAMB-Institute of Applied Microbiology, ABBt-Aachen Biology and Biotechnology, RWTH Aachen University, Worringerweg 1, 52074 Aachen, Germany; 2 Institute of Bio- and Geosciences IBG-1: Biotechnology, Forschungszentrum Jülich, 52425 Jülich, Germany

**Keywords:** Fermentation, pH control, *Ustilago cynodontis*, Process optimization, Product toxicity, Itaconic acid

## Abstract

**Background:**

*Ustilago cynodontis* ranks among the relatively unknown itaconate production organisms. In comparison to the well-known and established organisms like *Aspergillus terreus* and *Ustilago maydis*, genetic engineering and first optimizations for itaconate production were only recently developed for *U. cynodontis,* enabling metabolic and morphological engineering of this acid-tolerant organism for efficient itaconate production. These engineered strains were so far mostly characterized in small scale shaken cultures.

**Results:**

In pH-controlled fed-batch experiments an optimum pH of 3.6 could be determined for itaconate production in the morphology-engineered *U. cynodontis* Δ*fuz7.* With *U. cynodontis ∆fuz7*^*r*^ *∆cyp3*^*r*^ *P*_*etef*_*mttA P*_*ria1*_*ria1*, optimized for itaconate production through the deletion of an itaconate oxidase and overexpression of rate-limiting production steps, titers up to 82.9 ± 0.8 g L^−1^ were reached in a high-density pulsed fed-batch fermentation at this pH. The use of a constant glucose feed controlled by in-line glucose analysis increased the yield in the production phase to 0.61 g_ITA_ g_GLC_^−1^, which is 84% of the maximum theoretical pathway yield. Productivity could be improved to a maximum of 1.44 g L^−1^ h^−1^ and cell recycling was achieved by repeated-batch application.

**Conclusions:**

Here, we characterize engineered *U. cynodontis* strains in controlled bioreactors and optimize the fermentation process for itaconate production. The results obtained are discussed in a biotechnological context and show the great potential of *U. cynodontis* as an itaconate producing host.

## Background

Itaconic acid is an unsaturated dicarboxylic acid with two pK_a_ values at 3.8 and 5.5 Depending on the pH value, the undissociated form H_2_ITA, the single dissociated form HITA^−^ and the double dissociated form ITA^2−^ can exist [1, 2]. Further it contains a methylene group, and its functional groups are especially interesting for the polymer industry. Depending on the groups chosen for polymerization, products with different properties can be synthesized and used for different applications [3, 4]. In addition, itaconate is also gaining increasing visibility in the pharmaceutical sector [5]. *Aspergillus terreus*, *Ustilago maydis* and *Ustilago cynodontis* are known as good itaconate producing organisms [6–8]. The biochemical pathways and underlying gene clusters responsible for itaconate production in these organisms are well-studied [6, 9–12]. Since over 60 years *A. terreus* is used for itaconate production by surface or stirred tank fermentation [3, 13]. What exactly triggers itaconate production in *A. terreus*, and especially why it produces itaconate, is still unknown [8]. In general production is initiated at low pH-values [7, 14, 15 ]. After initiating efficient itaconate production, it could be shown that increasing pH-value can enhance itaconate titers, whereby the timing of the pH increase is important [2, 16]. By controlling the pH at 3.4 after the itaconate initiating phase, product titers up to 160 g L^−1^ could be achieved [2]. Further productivity could be increased by media optimization and pH-shift experiment to 1.15 g L^−1^ h^−1^ [16] and the highest reported yield with 0.72 g_ITA_ g_GLC_^−1^ was reached by optimizing oxygen transfer [17]. Following submerged fermentation with *A. terreus*, the itaconic acid is typically purified by repeated crystallization in industrial settings [18]. Although *A. terreus* is a highly efficient itaconate producer, some drawbacks exist for this host. One feature that causes high costs is the ability to grow as pellet or mycelia, respectively [19]. While pellet sizes between 0.1 to 0.5 mm resulted in the highest itaconate productivity [20], growing in mycelial form leads to a stop of itaconate production [2, 19]. This morphology is strongly influenced by media compositions. Currently molasses is used as carbon source to reduce costs. Concentrations of > 5 µg/L of impurities like manganese are known to induce mycelium formation, and this impure substrate must therefore be pretreated by ion exchange chromatography or ferrocyanide treatment [7, 21]. Alternatively, these morphology issues might also be suppressed by the addition of short-chain alcohols or copper, which are used in citric acid production processes [22]. These additional steps or medium components, however, make medium preparation more costly and control of the fermentation process more complex [22]. Also, the biochemical basis for their beneficial effect is not fully elucidated and further investigations are necessary [7]. Other factors such as pH- and shear stress can also induce mycelial growth and reduce itaconate production in *A. terreus* [8]. Beyond the manganese sensitivity, the morphology issue and the peculiarities of *Aspergillus* in general drastically reduce the process window, including the applicable pH range, the presence of solids and the tolerance towards other medium impurities. Thus, in order to achieve a breakthrough in this very mature process, we investigate the Ustilaginaceae as alternative unicellular hosts that avoid these morphological and process-related drawbacks [23].

Besides *A. terreus* many Ustilaginaceae are known to produce itaconate naturally [24, 25]. The most well studied member of this family is *U.* *maydis*. In wildtype *U. maydis,* itaconate production is initiated by nitrogen limitation [26] and production takes place above pH-values of 5.5 [24], although engineered strains can produce itaconate at lower pH [27]. While its yeast-like growth behavior is a benefit especially for production in a bioreactor, current values for titer, yield and productivity on glucose are far away from that what is published for *A.* *terreus* [6, 9]. *U.* *cynodontis* is another promising Ustilaginaceae which, however, displayed strong filamentous growth [24, 28]. Unlike *U. maydis, U. cynodontis* has a high tolerance towards low pH, which poses major benefits for itaconate production. Recently we could overcome the strong filamentous growth behavior under biotechnologically relevant conditions through the deletion of *fuz7,* encoding a MAPK protein involved in the regulation of tube formation and filamentous growth [11]. Further it was possible to increase itaconate production up to 6.5-fold compared to the wildtype by metabolic engineering, involving the deletion of P450 monooxygenase-encoding *cyp3*, the overexpression of *ria1* encoding the itaconate cluster regulator, and heterologous expression of the mitochondrial tricarboxylate transporter MttA from *A. terreus* [11]. In this study, we apply this optimized *U. cynodontis* strain in controlled bioreactors. The optimal pH value for itaconate production is determined, followed by process optimization to enhance itaconate production by different glucose feeding strategies and by repeated batch. By this means, we demonstrate the potential of *U. cynodontis* as alternative acid-tolerant itaconate producer with a stable yeast-like morphology.

## Results and discussion

### Influence of pH and yeast extract on itaconate production by engineered *U. cynodontis*

**Fig. 1:**
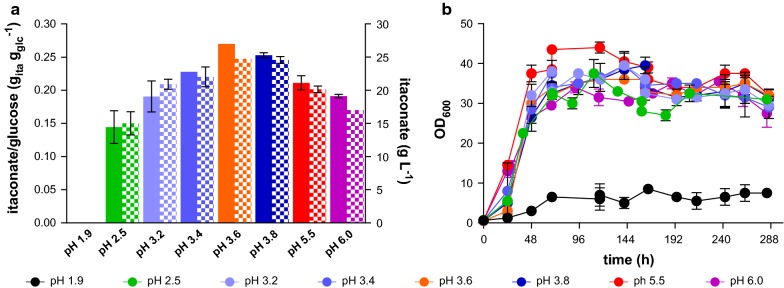
Controlled high-density pulsed fed-batch fermentation of *U. cynodontis* ∆*fuz7* at different pH values. **a** Yield in g_ITA_ g_GLC_^−1^ (filled bars) and itaconate concentration (patterned bars) and **b** OD_600_ during fermentation in a bioreactor containing batch medium without yeast extract with 50 g L^−1^ glucose, and 0.8 g L^−1^ NH_4_Cl, pulsed twice with 50 mL of a 50% glucose stock, controlled at different pH values titrated with NaOH. Error bars indicate the deviation from the mean (n = 2) with the exception of the fermentation at pH 3.6, which shows a single representative culture

Previously we reported that by deletion of *fuz7* the strong filamentous morphology of *Ustilago cynodontis* was switched to stable yeast like growth, resulting in a better production of itaconate. Shake flask experiments in different buffered media indicated that *U. cynodontis* has high acid tolerance, but the optimum for itaconate production could not be determined in this setup [11]. Since it is known that the pH is a key factor in itaconate production with considerable influence on later downstream processes, and that protonated itaconate leads to weak acid uncoupling [1, 2, 8, 16], we determined the optimal pH for itaconate production in *U. cynodontis* ∆*fuz7* by pH-controlled pulsed fed-batch fermentations (Fig. 1). Cultures were performed at pH values of 1.9, 2.5, 3.2, 3.4, 3.6, 3.8, 5.5 and 6.0, set from the beginning of inoculation by manual addition of HCl and controlled afterwards with NaOH. The stirrer was set to 1000 rpm in batch medium without yeast extract, 0.8 g L^−1^ NH_4_Cl and 50 g L^−1^ glucose, after which 50 ml of a 500 g/l glucose stock solution was pulsed twice when the concentration reached ± 30 g L^−1^. Corresponding titers, yields and OD_600_ are depicted in Fig. 1.

Strong growth inhibition was observed at pH 1.9 compared to the other cultures. However, *U. cynodontis* Δ*fuz7* both grew and produced itaconate at the second lowest pH of 2.5, although the yield was 1.9-fold lower than at the optimal pH of 3.6 where a titer of 24.7 g L^−1^ and a yield of 0.27 g_ITA_ g_GLC_^−1^ were reached. These differences in production are likely related to the higher level of weak acid stress. Below a pH value of 3.8 the protonated form (H_2_ITA) is predominant, which can diffuse through the plasma membrane and acidify the cytoplasm resulting in growth and/or product inhibition for the cell. In contrast, the dissociated forms HITA^−^ and ITA^2−^ cannot cross the membrane by diffusion due to their charge and stay in the fermentation broth [2, 29–31]. To determine the concentrations of each dissociation form of itaconic acid in this study CurTiPot was used [32, 33]. Between a pH value of 2.5 and 3.6 the concentration of protonated H_2_ITA at the end of the cultures was 14.6 ± 0.8 g L^−1^ (Table 1). This concentration is relatively constant, especially considering the much larger differences in total titer, indicating that this protonated product level is inhibitory for the cells. With further increasing pH H_2_ITA concentrations decrease and the relatively harmless dissociated forms become predominant, even though glucose was not fully consumed. Possibly, higher pH values change the regulation of the itaconate cluster genes. This was also observed in itaconic acid production in *A. terreus* where the optimum for production was determined at a pH of 3.4 [2]. However, with *A. terreus* a morphological change was the main reason for this decrease. Such a morphological change was excluded with *U. cynodontis* Δ*fuz7*. Likely, the pH optimum for itaconate production is at least in part governed by regulatory mechanisms of the genes in the itaconate cluster.

**Table 1:** Distribution of protonation states of itaconate in controlled high-density pulsed fed-batch fermentation of *U. cynodontis* ∆*fuz7* at different pH values

pH	H_2_ITA (g L^−1^)	HITA^−^ (g L^−1^)	ITA^2−^ (g L^−1^)	Total titer (g L^−1^)
1.9	0.0 ± 0.0	0.0 ± 0.0	0.0 ± 0.0	0.0 ± 0.0
2.5	14.1 ± 1.2	0.9 ± 0.1	0.0 ± 0.0	15.0 ± 1.2
3.2	15.9 ± 0.4	5.0 ± 0.1	0.1 ± 0.0	20.9 ± 0.5
3.4	14.6 ± 0.7	7.2 ± 0.3	0.1 ± 0.0	22.0 ± 1.1
3.6	13.7	10.7	0.3	24.7
3.8	10.7 ± 0.2	13.3 ± 0.2	0.6 ± 0.0	24.6 ± 0.4
5.5	0.1 ± 0.0	6.2 ± 0.1	13.8 ± 0.2	20.1 ± 0.4
6	0.0 ± 0.0	2.1 ± 0.0	14.9 ± 0.0	17.0 ± 0.0

Errors indicate the deviation from the mean (n = 2) with the exception of the fermentation at pH 3.6, which shows a single representative culture

For all used pH values no filamentous growth was observed, in accordance with previous observations [11]. Differences in the color of the fermentation broth were observed. While at low pH the fermenter broth was yellowish or white, at higher pH values it became more pigmented. Low amounts of erythritol as side product were measured which did not show any particular trend. Another major side product was (*S*)-2-hydroxyparaconate. It has a lower pK_a_-value than itaconate [34] and in *U. maydis*, low pH values stimulate the conversion of itaconate to (*S*)-2-hydroxyparaconate, likely by enabling passive itaconate re-uptake [9]. Through the deletion of *cyp3* (*S*)-2-hydroxyparaconate production could be abolished, leading to an increase in itaconate production. A further major increase was achieved by overexpression of *ria1* and *mttA*. Possibly, these modifications affect the pH optimum at which *U. cynodontis* produces itaconate, which should be investigated in the future.

**Fig. 2:**
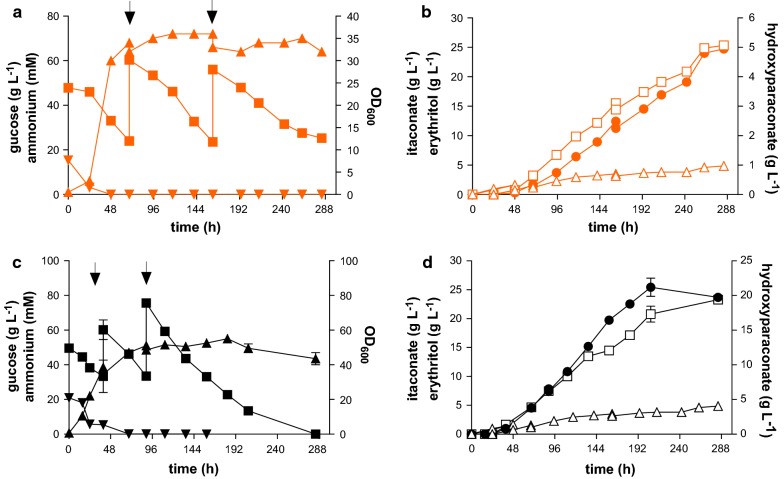
Controlled high-density pulsed fed-batch fermentation of *U. cynodontis* ∆*fuz7*. **a**, **c** OD_600_ (▲), glucose (■) and ammonium concentration (▼), **b**, **d** Concentration of itaconate (●), (*S*)-2-hydroxyparaconate (□) and erythritol (∆) during fermentation in a bioreactor containing batch medium without (**a**, **b**) or with yeast extract (**c**, **d**) with 50 g L^−1^ glucose, 0.8 g L^−1^ NH_4_Cl at pH 3.6 titrated with NaOH. Arrows indicate addition of 35 mL of a 50% glucose stock. Error bars indicate the deviation from the mean (n = 2) except for A and B, which show a single representative culture

The abovementioned determination of the pH optimum was performed in a fully mineral medium. Previous fermentations with *U. maydis* were often performed with 1 g L^−1^ yeast extract added to the starting medium [24]. To see if this addition influences production, fermentations were repeated with the same conditions at the determined optimal pH of 3.6, with the exception that this time 1 g L^−1^ yeast extract was not omitted from the batch medium (Fig. 2). The maximum titer of 25.5 ± 1.1 g L^−1^ itaconate and yield of 0.25 ± 0.01 g_ITA_ g_GLC_^−1^ were similar to those determined without yeast extract. In contrast maximum (*S*)-2-hydroxyparaconate production was increased by 3.5-fold to 17.3 ± 1.1 g L^−1^ and consequently the total acid concentration in batch medium with yeast extract was 1.4 fold higher. The key factor that could be improved in the case for itaconate was the productivity (Fig. 2b, d). Without yeast extract the total fermentation time was 288 h. Addition of yeast extract reduced this time to 206 h. This addition a complex medium component might increase the efforts for downstream purification, and the resulting rate gain should thus be considered in the context of the entire process [23]. For following experiments, full batch medium (which includes 1 g L^−1^ yeast extract) was used at a pH of 3.6.

### Enhanced itaconate production with optimized *U. cynodontis*

**Fig. 3:**
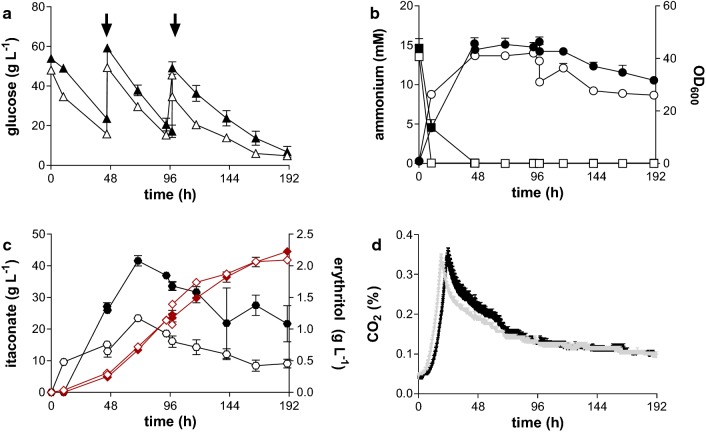
Controlled high-density fed-batch fermentation of *U. cynodontis* NBRC 9727 *∆fuz7*^r^ *∆cyp3*^r^ P_*etef*_*mttA* P_*ria1*_*ria1*; **a** glucose concentration; **b** OD_600_ (circles) and ammonium (squares) concentration; **c** itaconate (diamonds, red) and erythritol (circles) concentration and **d** CO_2_ off-gas concentration during fermentation in a bioreactor containing batch medium with 50 g L^−1^ glucose, 0.8 g L^−1^ NH_4_Cl with a natural pH shift from 6 to 3.6 (filled symbols/continuous grey line) or a constant pH of 3.6 (empty symbols/continuous black line) controlled with NaOH. Arrows indicate addition of 35 mL of 50% glucose. Error bars indicate the standard error of the mean (n = 3)

While pH optimum was determined with *U. cynodontis* ∆*fuz7*, the new hyperproducing strains described in Hosseinpour Tehrani et al. [11] were developed in parallel. The strain *U. cynodontis* ∆*fuz7*^r^ ∆*cyp3*^r^ *P*_*etef*_*mttA P*_*ria1*_*ria1* produces 6.5-fold more itaconate in shake flasks compared to the wildtype. In order to assess the performance of this new strain in controlled fed-batch fermentation, it was cultured in batch medium at a constant pH of 3.6. Cultures of *A. terreus* are often started at a more neutral pH, letting it drop during growth after which pH control is switched on [2, 16]. This pH shift can have a positive impact in the growth phase by reducing low-pH stress, but it may also pose a higher risk of bacterial contamination at industrial scale. To test the effect of such a pH shift on the engineered *U. cynodontis*, another fermentation was started at pH 6.0, letting the pH drop to 3.6, after which it was controlled at this level with NaOH (Fig. 3 and Table 2). As expected, significantly more itaconate was produced in these fed-batch fermentations compared to shaken batch cultures with the optimized ∆*fuz7*^r^ ∆*cyp3*^r^ *P*_*etef*_*mttA P*_*ria1*_*ria1* strain [11], and also compared to *U. cynodontis* ∆*fuz*7 in fed-batch cultures (Fig. 2).

**Table 2:** Itaconate production parameter of various fermentation conditions with *U. cynodontis* NBRC 9727 *∆fuz7*^r^ *∆cyp3*^r^ P_*etef*_*mttA* P_*ria1*_*ria1*

	Constant pH 3.6	pH shift 6–3.6	High nitrogen	Constant glucose feed
Titer (g L^−1^)^a^	44.5 ± 1.6	41.8 ± 0.3	83.0 ± 0.8	78.6
Y_P/S_ (g_ITA_ g_GLC_^−1^)^b^	0.39 ± 0.0	0.39 ± 0.0	0.30 ± 0.0	0.45
Y_P/S_^M^ (g_ITA_ g_GLC_^−1^)^c^	0.47 ± 0.0	0.44 ± 0.0	0.36 ± 0.0	0.61
r_p_ (g L^−1^ h^−1^)^d^	0.23 ± 0.0	0.21 ± 0.0	0.59 ± 0.0	0.42
r_p_^M^ (g L^−1^ h^−1^)^e^	0.38 ± 0.0	0.34 ± 0.0	1.4 ± 0.0	0.85

Errors indicate the standard error from the mean (n = 3) while fermentation with a constant glucose feed is a single representative culture

^a^Titer: maximum itaconate concentration

^b^Y_P/S_: overall yield of itaconate per consumed glucose

^c^Y_P/S_^M^: maximum yield during the production phase

^d^r_p_: overall production rate

^e^r_p_^M^: maximum production rate

The growth phase (derived from offgas CO_2_ values) was approximately 5 h shorter when starting at pH 6, but in spite of this, the constant pH of 3.6 had no negative impact on production parameters. On the contrary, the maximum titer of 44.5 ± 1.6 g L^−1^ in the fermentation with a constant pH of 3.6 culture was slightly, but not significantly, higher than that of the fermentation with the pH shift with 41.8 ± 0.3 g L^−1^ (Fig. 3c, Table 2). Also similar values for biomass and CO_2_ formation were observed for both conditions (Table 3, Fig. 3b, d). Further, nitrogen limitation was achieved faster in fermentation with constant pH (Fig. 3b). For both conditions the same yield was observed (Table 2). The carbon balance of all tested conditions is closed to within 95% (Table 3). Some unidentified components such as ustilagic acid [24] may be produced, and should be investigated in the future.

**Table 3:** Carbon distribution of various fermentation conditions with *U. cynodontis* NBRC 9727 *∆fuz7*^r^ *∆cyp3*^r^ P_*etef*_*mttA* P_*ria1*_*ria1* in batch medium with various glucose and NH_4_Cl concentrations

	Constant pH 3.6	pH shift 6–3.6	High nitrogen
Itaconate (%)	51.6 ± 0.7	50.7 ± 1.3	38.3 ± 0.7
CO_2_ (%)	25.4 ± 2.2	25.9 ± 1.9	28.8 ± 0.6
CDW (%)	19.9 ± 2.3	20.9 ± 0.8	26.0 ± 1.0
Erythritol (%)	1.0 ± 0.4	0.4 ± 0.1	2.2 ± 0.1
Mass balance (%)	97.8 ± 1.9	97.9 ± 2.2	95.3 ± 2.0

Errors indicate the standard error of the mean (n = 3)

Overall, the production parameters achieved with a constant pH of 3.6 were similar to those achieved with *U. maydis* at pH > 6 where a maximum titer of 54.8 ± 2.8 g L^−1^, productivity of 0.33 ± 0.02 g L^−1^ h^−1^ and a yield of 0.48 ± 0.02 g_ITA_ g_GLC_^−1^ were reached [9]. Depending on the process setup, the low pH optimum of *U. cynodontis* can provide significant benefits for downstream processing [35, 36]. Also, the lower pH reduces the risk of contamination [1], possibly enabling auto-sterile conditions, although this is not given even for low pH processes [37]. Given these advantages and the fact that no differences in itaconate production were observed, further fermentations were performed at a constant pH of 3.6. However, it should be considered that the pH optimum for the production strain *U. cynodontis* ∆*fuz7*^r^ ∆*cyp3*^r^ *P*_*etef*_*mttA P*_*ria1*_*ria1* may have shifted. The elimination of (*S*)-2-hydroxyparaconate, a monocarboxylate with a lower pKa value than itaconate [34], will alter acidification. Also, pH plays a role in the induction of itaconate production in *A. terreus* [2], making it plausible that the overexpression of the itaconate cluster regulator Ria1 affects induction of itaconate production in relation to pH in *U. maydis*. These putative effects will be subject to future study.

Previous high-density fermentations with *U. maydis* have resulted in higher titers and productivities [9, 38], potentially reducing process and investment costs in an industrial context [39, 40]. However, they often come at a cost of lower yields, although the relation between cell density and production yield, titer, and rate are often non-linear [41]. In order to investigate the effect of higher cell densities of *U. cynodontis ∆fuz7*^r^ *∆cyp3*^r^ *P*_*etef*_*mttA P*_*ria1*_*ria1*, fermentations with 200 g L^−1^ glucose and 4 g L^−1^ NH_4_Cl in the batch medium were performed. With this change, however, it must also be taken into account that problems can arise such as limitation and/or inhibition of substrates, high evolution rates of CO_2_ and heat, high oxygen demand, and increased viscosity of the medium [42].

**Fig. 4:**
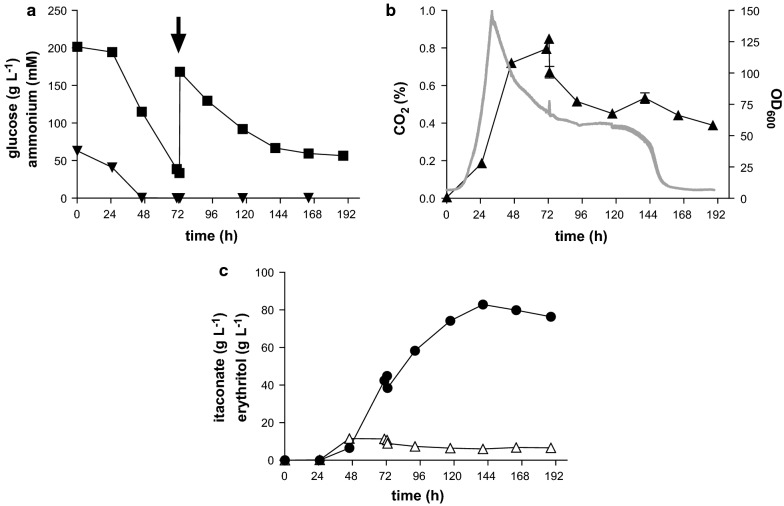
Controlled high density fed-batch fermentation of *U. cynodontis* NBRC 9727 *∆fuz7*^*r*^ *∆cyp3*^*r*^ P_*etef*_*mttA* P_*ria1*_*ria1.* glucose (■) and ammonium (▼) concentration; **b** CO_2_ concentration (continuous line) and OD_600_ (▲); **c** itaconate (●) and erythritol (∆) concentration during fermentation in a bioreactor containing batch medium with 200 g L^−1^ glucose, 4.0 g L^−1^ NH_4_Cl at pH 3.6 titrated with NaOH. The arrow indicates addition of 100 mL of 50% glucose. Error bars indicate the standard error from the mean (n = 3)

The fivefold increase in ammonium as growth-limiting nutrient resulted in a maximum titer of 82.9 ± 0.8 g L^−1^ itaconate after 140 h. This maximum was followed by a gradual decrease of itaconate, even though glucose was still present (Fig. 4). Simultaneously, the CO_2_ concentration in the exhaust gas dropped from 0.4% to 0.04% and glucose consumption stopped (Fig. 4a, b). A maximum productivity of 1.44 ± 0.02 g L^−1^ h^−1^ was reached between 46 and 73 h, which is 3.8-fold more compared to fermentation with low nitrogen content (Table 3).

**Fig. 5:**
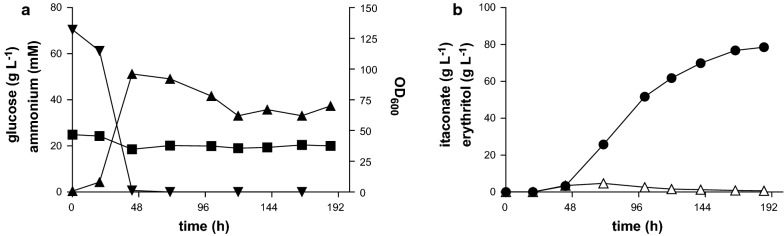
Controlled high density fed-batch fermentation of *U. cynodontis* NBRC 9727 *∆fuz7*^r^ *∆cyp3*^r^ P_*etef*_*mttA* P_*ria1*_*ria1*. **a** Glucose (■) and ammonium (▼) concentration and OD_600_ (▲); and **b** itaconate (●) and erythritol (∆) concentration during a single representative bioreactor cultivation in batch medium with constant glucose concentration, 4.0 g L^−1^ NH_4_Cl at pH 3.6 titrated with NaOH

Interestingly, although five times more nitrogen was used, the OD_600_ was only three times higher compared to the fermentation with 0.8 g L^−1^ NH_4_Cl, suggesting a possible limitation in other medium components like was observed in *U. maydis* [43]. Further Klement et al. [43] and Zambanini et al. [44] could show that inhibition by high NH_4_Cl concentrations affect biomass growth, which can be avoided by pulse-feeding the nitrogen source. A gradual decrease in productivity and biomass concentration is visible after 72 h indicating cell stress was initiated at this time point. The relatively sudden drop in the CO_2_ evolution rate at 140 h indicates that at this point a critical product concentration is reached at which the cells are unable to maintain their vigor, likely because at this point they are unable to counteract weak acids uncoupling due to the reduced substrate uptake rate. It is known that itaconate can inhibit isocitrate-lyase and fructose-6-phosphate 2-kinase, as well as substrate phosphorylation in mitochondria [45–47], which may further contribute to lowering the substrate uptake rate. With wildtype *U. maydis* external addition of 80 g L^−1^ itaconate fully inhibits its production [43]. Similar experiments should be performed with these engineered *U. cynodontis* strains to test the abovementioned hypothesis of product inhibition. In general, the high cell density cultures significantly increased the maximum titer and productivity compared to the low cell density cultures, at a relatively small cost to the product yield (Fig. 4 and Table 2). The pulsed feed in the abovementioned high-density culture coincides with a significant drop in the production rate, likely due to cumulative osmotic and weak acid stress. In addition, it is known for Ustilaginaceae that high a glucose concentration leads to slower growth and osmotic stress [9]. For these reasons, a fermentation with a constant glucose concentration of 20 g L^−1^ was performed whereby other parameters were equivalent to the fermentation with high nitrogen. In order to ensure a constant substrate concentration, an in-line system for the analysis of glucose from Trace Analytics (Braunschweig, Germany) was used. Cell-free in-line sampling was enabled by a dialysis probe. The glucose measurement itself is based on an enzymatic reaction with glucose oxidase [33]. The inline System of TraceAnalytics was connected to the BioFlo 120^®^ system (Eppendorf, Jülich, Germany), enabling it to be coupled to a pump which regulates the glucose feed depending on the measured glucose concentration. Thus the glucose uptake rate could be determined by the rate of the pump. As an additional control, the consumption of the glucose stock solution was measured by weighing. Using this setup, nearly the same titer could be reached compared to the equivalent fermentation with pulsed glucose feeds, however, with a lower overall (0.42 g L^−1^ h^−1^) and maximum (0.84 g L^−1^ h^−1^) production rate (Fig. 5 and Table 2). In contrast, glucose consumption was reduced by 30% compared to the pulsed fed batch (Additional file 1: Fig. S1) leading to a much higher overall yield of 0.41 g_ITA_ g_GLC_^−1^. During the production phase between 43 and 186 h, a yield of 0.61 g_ITA_ g_GLC_^−1^ was achieved, which is 84% of the theoretical maximum pathway yield. This much higher yield, along with the lower erythritol formation, strongly indicates that the cells suffer less from osmotic stress compared to the pulsed fed batch. This is also corroborated by the decrease in productivity upon the pulse in the fermentation with high nitrogen, where the combined stress of substrate and product concentrations is about fourfold higher. Despite the improvements achieved with the constant glucose concentration, the problem of product toxicity remains. Itaconate production and glucose uptake rates decreased above 50 g L^−1^, as also observed in the pulsed fed batch (Additional file 1: Fig. S1).

**Fig. 6:**
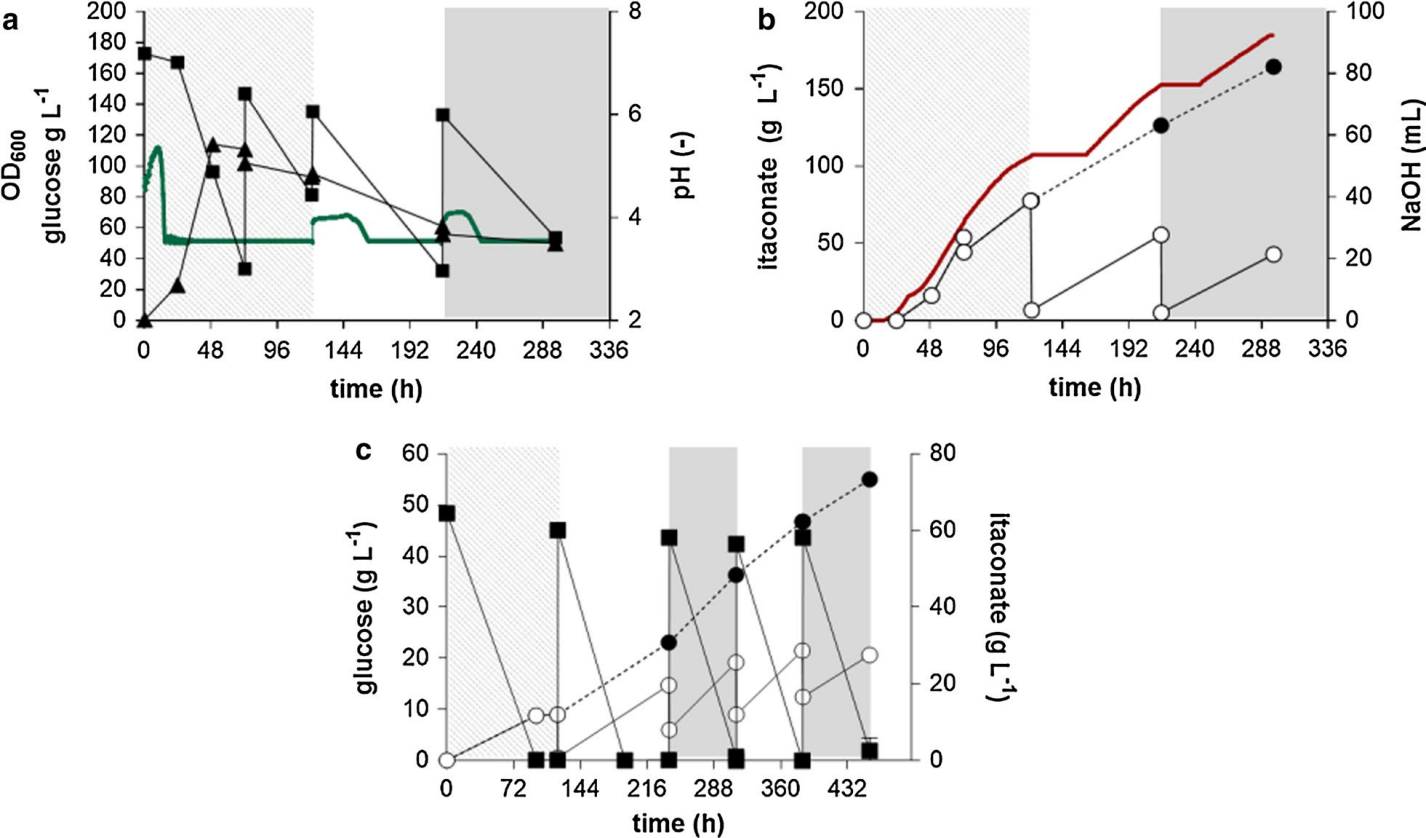
Repeated batch with cell recycling of *U. cynodontis* NBRC 9727 *∆fuz7*^r^ *∆cyp3*^r^ P_*etef*_*mttA* P_*ria1*_*ria1*. **a** OD_600_ (▲) and pH (green line); **b** glucose (■) itaconate (●) and total itaconate (○, dashed line) concentration, and used NaOH (red line) during a single representative fermentation in a bioreactor containing batch medium with 200 g L^−1^ glucose, 4 g L^−1^ NH_4_Cl at pH 3.6 titrated with 10 M NaOH (**a**, **b**), or in shake flasks containing screening medium with 33 g L^−1^ CaCO_3_ and 50 g L^−1^ glucose (**c**). For the repeated batches (indicated by grey shading), culture broth was centrifuged and the biomass was subsequently re-suspended in 0.5 L fresh batch medium without nitrogen, 170 g L^−1^ glucose and 0.5 g L yeast extract (**a**, **b**), or culture broth was centrifuged and the biomass was subsequently re-suspended in screening medium without nitrogen, 50 g L^−1^ glucose and 25 g L^−1^ CaCO_3_ (**c**). Error bars indicate the standard error from the mean (n = 4)

Both fermentation approaches (pulsed fed batch and constant glucose concentration) show clear signs of product toxicity at around 80 g L^−1^ itaconate at a pH of 3.6 (Figs. 4, 5, 6 and Table 2). One way to overcome this would be in situ product removal by calcium salt precipitation as shown for *U. maydis* [48] and  *U. vetiveriae* [44] or by reactive extraction methods [49]. Alternatively, a continuous or semi-continuous process with cell recycling can help to overcome product toxicity as well, by extending the productive time of the biomass [50].

### Extension of productive time by repeated batch fermentation

In order to assess the stability of the biocatalyst under low pH, and to overcome product toxicity, a repeated batch approach with cell recycling was applied. The same conditions as in the pulsed fed-batch fermentation with 4.0 g L^−1^ NH_4_Cl were used, but after 120 h, cells were centrifuged and resuspended in fresh batch medium without NH_4_Cl. Yeast extract (0.5 g L^−1^) was added to the medium because this addition greatly improved cell recovery in initial pilot experiments. In the initial batch phase, 77.6 g L^−1^ itaconate was produced which corresponds to a yield of 0.4 g_ITA_ g_GLC_^−1^ (Fig. 6). In the first repeated batch phase 49 g L^−1^ itaconate with a yield of 0.5 g_ITA_ g_GLC_^−1^ and in the second repeated batch 38 g L^−1^ with a yield of 0.5 g_ITA_ g_GLC_^−1^ were produced. Base totalizer revealed that in the first 39 h of the first repeated batch and 28 h of the second batch, a lag phase occurred in which no itaconate was produced (Fig. 6b). This lag phase can likely be attributed to the centrifugation steps used for the cell recycling, which deprive the cells of oxygen under low pH conditions. This lag phase was absent in shake flasks with CaCO_3_ at neutral pH (Fig. 6c) and in addition no yeast extract was necessary in shake flask to allow the cells to recover after the centrifuging step. The difference in the lag phases may be explained by different centrifugation times. While the first batch was centrifuged for 20 min, the second batch was only centrifuged for 5 min to minimize oxygen limitation. Cell density decreased with each repeated batch, which was reflected in the volumetric productivity. Overall, the cell recycling positively affected the product yield, which was stable across two repeated batches. However, significant lag phases and reductions in biomass and production rates indicate a high stress imposed by the centrifugation steps applied here for cell recycling. To overcome these issues a membrane-based cell retention system should be used [51].

## Conclusions

This study demonstrates the applicability of the pH tolerant *Ustilago cynodontis* in controlled fed-batch cultivations, reaching high yield, titer and rate at a low pH value. High density fermentation, especially coupled with a continuous glucose feed, provided the overall best balance of production parameters, reaching high titers and yields with a minimal loss in productivity. Titers of up to 82.9 g L^−1^ were reached, which imposed significant product toxicity onto the cell, completely inhibiting the substrate uptake rate. Repeated-batch cultures indicated a high stability of the biomass, showing the potential to overcome product toxicity in a continuous itaconate production system with cell retention, especially if centrifugation steps can be avoided in the future. In all, this study demonstrates the possibilities enabled by the stable yeast-like morphology of the engineered *U. cynodontis* strain, while retaining the benefit of low pH fermentation for itaconic acid production.

## Methods

### Strains and culture conditions

Controlled batch cultivations were performed with *Ustilago cynodontis ∆fuz7* and *U. cynodontis ∆fuz7*^*r*^ *∆cyp3*^*r*^ P_*etef*_*mttA* P_*ria1*_*ria1* in a BioFlo^®^ 115 bioreactor (Eppendorf, Germany) with a total volume of 1.3 L and a working volume of 0.5 L. The Eppendorf BioFlo^®^ 120 bioprocess control station (Eppendorf, Germany) was used in combination with the online glucose measurement system from Trace Analytics (Trace Analytics, Germany) with a total volume of 2.0 L and a starting volume of 1.0 L. All cultivations were performed in batch medium according to Geiser et al. [9] containing 0.2 g L^−1^ MgSO_4_·7H_2_O, 0.01 g L^−1^ FeSO_4_·7H_2_O, 0.5 g L^−1^ KH_2_PO_4_, 1 g L^−1^ yeast extract (Merck Millipore, Germany) 1 mL L^−1^ vitamin solution, and 1 ml L^−1^ trace element solution and varying concentrations of glucose and NH_4_Cl as indicated. The vitamin solution contained (per liter) 0.05 g d-biotin, 1 g d-calcium panthotenate, 1 g nicotinic acid, 25 g myo-inositol, 1 g thiamine hydrochloride, 1 g pyridoxol hydrochloride, and 0.2 g para-aminobenzoic acid. The trace element solution contained (per liter) 15 g EDTA, 0.45 g of ZnSO_4_∙7H_2_O, 0.10 g of MnCl2∙4H_2_O, 0.03 g of CoCl_2_∙6H_2_O, 0.03 g of CuSO_4_∙5H_2_O, 0.04 g of Na_2_MoO_4_∙2H_2_O, 0.45 g of CaCl_2_∙2H_2_O, 0.3 g of FeSO_4_∙7H_2_O, 0.10 g of H_3_BO_3_, and 0.01 g of KI. During cultivation, pH 1.9, 2.5, 3.2, 3.4, 3.6, 3.8, 5.5 and 6.0 were maintained by automatic addition of 10 M NaOH and stirring rate was constant at 1000 rpm. For repeated-batch, the culture was centrifuged for 5 min to 20 min at 80 g and afterwards re-suspended in 0.5 L batch medium without NH_4_CL and 0.5 g L^−1^ yeast extract. The bioreactor was aerated with an aeration rate of 1 L min^−1^ (2 vvm) for working volume of 0.5 L or 2 L min^−1^ (1 vvm) for total volume of 2 L, while evaporation was limited by sparging the air through a water bottle. The bioreactor was inoculated to a final OD_600_ of 0.75 with cells from an overnight culture in 50 mL screening medium according to [24] containing 50 g L^−1^ glucose and 100 mM MES buffer. Repeated batch cultivation in shake flask cultivation was performed in screening medium according to Geiser et al. [24] containing 33 g L^−1^ CaCO_3_ and 50 g L^−1^ glucose. The cultures were centrifuged at 1473 g for 5 min at 30 °C with a Heraeus Megafuge 16R (Thermo Scientific) and a TX-400 rotor (Thermo Scietific). For subsequent cultivation, the cells were re-suspended in screening medium containing 25 g L^−1^ CaCO_3_ without NH_4_Cl and 50 g L^−1^ glucose.

### Analytical methods

Cell densities were measured by determining the absorption at 600 nm with an Ultrospec 10 Cell Density Meter (Amersham Biosciences, Chalfont St Giles, UK).

For CDW determination 1 mL culture broth was centrifuged at maximum speed (Heraeus Megafuge 16R, TX-400 rotor, Thermo Scientific) and pellet was lyophilized (Scan Speed 40 lyophilizer, Labogene ApS) for 24 h at 38 °C and weighed afterwards.

Off-gas analysis for online monitoring of CO_2_ content were performed with BCpreFerm sensors (BlueSens gas sensor GmbH). The online CO_2_ signal (%) was converted into moles using a molar volume of 24 L mol^−1^. Mass balancing was achieved by subtracting the C-mol amount of biomass, off-gas and products (itaconate, erythritol), from the substrate glucose. For biomass, a carbon content of 57.9% (w/w) was assumed based on the biomass compositions of *U. maydis* under nitrogen limitation [43]. Differential interference contrast (DIC) microscopy was performed with a Leica DM500 light microscope (Leica Microsystems). Images were recorded with a Leica ICC50 digital microscope camera (Leica Microsystems). Images were taken at 630-fold magnification. The cell morphology was analyzed by microscopy at different time points in all cultivations.

The ammonium concentration in the culture supernatant was measured by a colorimetric method according to [52] using salicylate and nitroprusside.

Products in the supernatants were analyzed in a DIONEX UltiMate 3000 High Performance Liquid Chromatography System (Thermo Scientific, Germany) with an ISERA Metab AAC column 300 × 7.8 mm column (ISERA, Germany). As solvent 5 mM H_2_SO_4_ with a flow rate of 0.6 mL min^−1^ and a temperature of 40 °C was used. Samples were filtered with Rotilabo^®^ (CA, 0.20 µm, Ø 15 mm) or Acrodisc^®^ (GHP 0.20 µm, Ø 13 mm) syringe filters and afterwards diluted up to 1:30 with 5 mM H_2_SO_4_. Itaconate, (*S*)-2-hydroxyparaconate and erythritol, were determined with a DIONEX UltiMate 3000 Variable Wavelength Detector set to 210 nm, and glucose with a refractive index detector SHODEX RI-101 (Showa Denko Europe GmbH, Germany). Analytes were identified via retention time and UV/RI quotient compared to corresponding standards. Additionally, presence of glucose was verified with glucose test-stripes from Macherey–Nagel. For (*S*)-2-hydroxyparaconate standards, samples of previous studies were used, where (*S*)-2-hydroxyparaconate was synthesized and purified [9]. Since the purity (~ 70%) of these samples is not exactly known, indicated (*S*)-2-hydroxyparaconate values should be taken as rough estimates only.

All values are the arithmetic mean of at least two biological replicates otherwise it is indicated. Error bars indicate the deviation from the mean for n = 2, if n > 2 error bars indicate the standard error of the mean. Statistical significance was assessed by *t* test (two-tailed distribution, heteroscedastic, p ≤ 0.05).

## Supplementary information

**Additional file 1.** Glucose consumption of fermentations in batch medium with pulsed or constant feed.

## Data Availability

All data generated or analysed during this study are included in this published article and its Additional files.
