# Supplementary material for: Process engineering of pH tolerant Ustilago cynodontis for efficient itaconic acid production
Source: Microb Cell Fact. 2019 Dec 12;18:213. doi: 10.1186/s12934-019-1266-y (PMC6909570; doi:10.1186/s12934-019-1266-y)
Supplement: Supplementary file 1 — Additional file 1. Glucose consumption of fermentations in batch medium with pulsed or constant feed. [file 12934_2019_1266_MOESM1_ESM.docx]

Additional data to:

**Process engineering of pH tolerant *Ustilago cynodontis* for efficient itaconic acid production**

Hamed Hosseinpour Tehrani^1^, Katharina Saur^1^, Apilaasha Tharmasothirajan^1^, Lars M. Blank^1^ & Nick Wierckx*^1, 2^

^‡^These authors contributed equally to this manuscript

*to whom correspondence should be addressed

^1^ iAMB – Institute of Applied Microbiology, ABBt – Aachen Biology and Biotechnology, RWTH Aachen University, Worringerweg 1, 52074 Aachen, Germany

^2^ Institute of Bio- and Geosciences IBG-1: Biotechnology, Forschungszentrum Jülich, 52425 Jülich, Germany

Address for correspondence:

Prof. Dr. Nick Wierckx

Phone: +49 246161 85247

Fax: +49 246161 2710

email: n.wierckx@fz-juelich.de

**Additional figures**

Fig. S1. Glucose consumption of *U. cynodontis* NBRC 9727 *∆fuz7*^r^ *∆cyp3^r^* P*_etef_mttA* P*_ria1_ria1*. Glucose consumption of fermentation in batch medium with pulsed-feed (●) or constant glucose concentration (continuous line), 4.0 g L^-1^ NH_4_Cl at pH 3.6 titrated with NaOH. Error bars indicate the standard error of the mean (n=3) while fermentation with a constant glucose feed is a single representative culture.
